# Comprehensive Phenolic and Free Amino Acid Analysis of Rosemary Infusions: Influence on the Antioxidant Potential

**DOI:** 10.3390/antiox10030500

**Published:** 2021-03-23

**Authors:** Juliana A. Barreto Peixoto, Gerardo Álvarez-Rivera, Rita C. Alves, Anabela S. G. Costa, Susana Machado, Alejandro Cifuentes, Elena Ibáñez, M. Beatriz P. P. Oliveira

**Affiliations:** 1 REQUIMTE/LAQV, Department of Chemical Sciences, Faculty of Pharmacy, University of Porto, R. J. Viterbo Ferreira, 4050-313 Porto, Portugal; jpeixoto@ff.up.pt (J.A.B.P.); acosta@ff.up.pt (A.S.G.C.); su_tche@hotmail.com (S.M.); beatoliv@ff.up.pt (M.B.P.P.O.); 2 Laboratory of Foodomics, Institute of Food Science Research, CIAL, CSIC, Nicolas Cabrera 9, 28049 Madrid, Spain; gerardo.alvarez@csic.es (G.Á.-R.); a.cifuentes@csic.es (A.C.); elena.ibanez@csic.es (E.I.)

**Keywords:** *Rosmarinus officinalis* L., bioactive compounds, antioxidants, amino acids, UHPLC-ESI-QTOF-MS, RP-HPLC-FLD

## Abstract

The phenolics profile, free amino acids composition, and antioxidant potential of rosemary infusions were studied. Forty-four compounds belonging to nine different groups (hydroxybenzoic acids, hydroxycinnamic acids, flavan-3-ols, flavanones, flavones, phenolic diterpenes, hydroxybenzaldehydes, coumarins, and pyranochromanones) were identified by UHPLC-ESI-Q-TOF-MS. Of these, seven were firstly described in rosemary infusions: a rosmanol derivative, two dihydroxycoumarin hexosides, a hydroxybenzaldehyde, a dihydroxybenzoic acid hexoside, coumaric acid hexoside, and isocalolongic acid. The free amino acid profile of the beverages was also reported by the first time with seven amino acids found (asparagine, threonine, alanine, tyrosine, phenylalanine, isoleucine, and proline). Furthermore, DPPH^•^ scavenging ability, Ferric Reducing Antioxidant Power and Oxygen Radical Absorbance Capacity, as well as total phenolics and flavonoids contents, were assessed. Overall, rosemary infusions showed to be a very good source of antioxidants. A 200 mL cup of this infusion contributes to the ingestion of ~30 mg of phenolic compounds and about 0.5–1.1 μg of free amino acids. This type of beverages may present a positive impact on the maintenance of the body antioxidant status and contribute to the prevention of oxidative stress related diseases.

## 1. Introduction

Rosemary (*Rosmarinus officinalis* L., Lamiaceae family) is quite appreciated in worldwide gastronomy due to its aromatic flavor and preservative properties [1,2,3,4]. In addition, this plant is also traditionally consumed in the infusion form aiming at the treatment and prevention of several health disorders, such as dyspepsia, mild spasmodic disorders, headache, depression, anxiety, respiratory disorders, minor peripheral circulatory disorders, and minor muscular and articular pain [1,3,5,6,7,8]. Most of these properties and, in particular, the antioxidant ones, are mainly attributed to the phenolic acids, phenolic diterpenes, and flavonoids present on rosemary [2,3,4,9,10]. Actually, the antioxidant activity is on the basis of the numerous properties attributed to these phenolic compounds, as they can act by different mechanisms of action, namely by free radical scavenging, hydrogen donation, metal ion chelation, by stimulating the cell antioxidant defenses, among others [11,12,13]. This versatility of mechanisms supports the increasing number of studies demonstrating that their regular consumption, in the necessary amounts, may help to prevent the generation of reactive oxygen species (ROS) that can cause pathological oxidative damages to cells and, consequently, contribute to the development of chronic diseases [11,12,14].

Beyond phenolic compounds, plants are also endowed by many other antioxidants that contribute to the biological properties of plants and increase the activity of phenolics through possible synergisms [3,4,15]. For example, some amino acids, besides playing a key role in many physiological functions (e.g., constituents of proteins and peptides, precursors of important metabolites, nitrogen storage molecules, regulators of important metabolic pathways, etc.), can also act as efficient antioxidants [15,16,17]. For example, the aromatic (tryptophan, phenylalanine, and tyrosine) and the acidic (aspartate and glutamate) ones, as well as asparagine, cysteine, alanine, isoleucine, methionine and proline can help to decrease the oxidative stress by several mechanisms, namely by scavenging free radicals, reduction of hydroperoxides, chelation of pro-oxidative transition metals, or even by acting as oxidative stress biomarkers [15,16,18,19]. Moreover, amino acids are precursors and constituents of important antioxidants peptides (e.g., glutathione) and enzymes (e.g., glutathione peroxidase, glutathione reductase, catalase, and superoxide dismutase) and particularly in plants they are essential for the biosynthesis of important bioactive compounds, such as phenolics and glucosinolates [15,16,18]. Therefore, the amino acid composition of plants should also be an important object of study, since this can add even more value to plants like rosemary with bioactive properties, opening the door to novel studies.

Although there are numerous studies on the bioactive properties of rosemary, many of them intend to characterize the plant itself or to evaluate/compare extraction techniques [2,9,10,20,21], and not so much to characterize the way the plant is often consumed by population—the infusion form. Furthermore, there is a lack of knowledge about the free amino acid composition of rosemary, particularly in the form of infusion, as well as its impact on the antioxidant properties of this beverage.

In this way, this work aimed to investigate the influence of phenolics, flavonoids, and free amino acids on the antioxidant activity of rosemary infusions. The phenolics and free amino acids profiles of the beverages were drawn to understand which compounds present in the beverages could contribute to antioxidant activity. As far as it is known, the free amino acids composition of rosemary infusions is reported for the first time.

## 2. Materials and Methods

### 2.1. Reagents and Standards

The following reagents and standards were from Sigma-Aldrich (St. Louis, MO, USA): 1,1-diphenyl-2-picrylhydrazyl radical (DPPH^•^), 2,4,6-tripyridyl-s-triazine (TPTZ), 2,2′-azo-bis,2-amidinopropane dihydrochloride (AAPH), ferric chloride, sodium fluorescein, ferrous sulfate, AlCl_3_, trolox, p-coumaric acid, caffeic acid, catechin, carnosic acid, chlorogenic acid, luteolin-7-*O*-glucoside, gallic acid, rosmarinic acid, rutin, syringic acid, vanillic acid, l-aspartic acid, l-glutamic acid, l-asparagine, l-serine, l-glutamine, l-histidine monohydrochloride monohydrate, glycine, l-threonine, l-arginine monohydrochloride, l-alanine, l-tyrosine, l-valine, l-leucine, l-lysine monohydrochloride, l-methionine, l-tryptophan, l-phenylalanine, l-isoleucine, *trans*-4-hydroxy-l-proline, l-proline, l-norvaline and the Amino Acids Mix Solution (Trace CERT^®^). The Folin-Ciocalteu’s reagent, protocatechuic acid, 4-hydroxybenzoic acid, hesperidin, hesperetin, and genkwanin were all from Merck (Darmstadt, Germany). HPLC grade methanol and acetonitrile, formic acid, and sodium azide (99%) were purchased from Honeywell Riedel-de Haën (Germany). Borate buffer (pH 10.2), *o*-phtalaldedehyde/3-mercaptopropionic acid (OPA/3-MPA), and 9-fluorenylmethyl chloroformate (FMOC) were from Agilent Technologies (Santa Clara, CA, USA). Ultrapure water was prepared in a Millipore System (Bedford, MA, USA). The other reagents were of analytical grade.

### 2.2. Samples

Rosemary leaves (*Rosmarinus officinalis* L., folium) from four different commercial brands were acquired from two herbalists (brands B and D) and two supermarkets (brands A and C). To guarantee the consistency of results, the selected products had the same form of presentation (dried rosemary leaves, with similar-sized fragments) and were intended for consumption as infusion. All the products were from Portugal. However, one of the commercial brands selected presented their products available in bulk, while the others were available in packages. The plants were stored, until infusions’ preparation, protected from the light and at room temperature.

### 2.3. Preparation of Rosemary Infusions

The beverages were prepared according to manufacturers’ recommendations to mimic the way they are generally consumed by population. Briefly, 1 g of leaves were mixed with 200 mL of boiling water and left resting for 5 min, with two agitations during this period. The infusions were then filtered, and aliquots were taken and stored at −21 °C until analyses. For each commercial brand, infusions were prepared in triplicate.

### 2.4. Study of the Infusions’ Antioxidant Properties 

The antioxidant activity of the infusions (assessed by DPPH^•^ scavenging ability, ferric reducing antioxidant power (FRAP), and oxygen radical absorbance capacity (ORAC) assays), as well as the total contents in phenolics and flavonoids were spectrophotometrically determined using a Synergy HT microplate reader (Biotek Instruments, Inc., GENS5, Winooski, VT, USA). 

#### 2.4.1. DPPH^•^ Inhibition

The DPPH^•^ inhibition of the different infusions was determined as previously described [22], with minor adaptations. In brief, a sample aliquot (30 μL) reacted during 20 min with 270 µL of DPPH^•^ in ethanol (6 × 10^−5^ M). The absorbance decrease was then measured at 525 nm. A calibration curve was prepared with Trolox (linearity range: 5–175 mg/L; R^2^ = 0.996), and the results were presented as µg of Trolox equivalents (TE) per mL of infusion. The assay was executed in triplicate.

#### 2.4.2. Ferric Reducing Antioxidant Power (FRAP)

The reducing power of the samples was assessed as previously described [22]. Briefly, an aliquot (35 μL) of the diluted sample (1:10) reacted with 265 µL of FRAP reagent (freshly prepared by mixing 10 mL of acetate buffer (0.3 M), 1 mL of TPTZ solution (10 mM), and 1 mL of ferric chloride (20 mM)) and, after incubation (37 °C, 30 min), the increase of absorbance was measured at 595 nm. A calibration curve was prepared with ferrous sulfate (linearity range: 25–500 µM; R^2^ = 0.9996) and the results were presented as µg of ferrous sulfate equivalents (FSE) per mL of infusion. The assay was performed in triplicate.

#### 2.4.3. Oxygen Radical Absorbance Capacity (ORAC)

The ability of the different infusions to act as scavengers against peroxyl radical (ROO^•^) was evaluated according to Peixoto et al. [23]. Very briefly, a diluted sample (1:10) aliquot (25 μL) was mixed with fluorescein (61 nM), followed by a 30 min incubation at 37 °C. Then, 25 μL of AAPH (19 nM) was added immediately before starting fluorescence measurements (λ_exc_ = 480 nm and λ_em_ = 520 nm), which were carried out every 2 min, during 2 h, at 37 °C. The net area under the curve (AUC) of the standard (Trolox) and samples were calculated and a calibration curve was obtained by plotting the net AUC of different concentrations of the standard against the average net AUC of the measurements for each concentration (linearity range: 1.57–50 µg/mL; R^2^ = 0.9996). The results were presented as µg of Trolox equivalents (TE) per mL of infusion. The procedure was performed in triplicate.

#### 2.4.4. Total Phenolics Content

The total content of phenolic compounds in the different infusions was estimated according to Nunes et al. [24]. Briefly, a sample aliquot (30 μL) was mixed with 150 µL of Folin-Ciocalteu reagent (1:10) and 120 µL of 7.5% Na_2_CO_3_. The mixture was then subjected to two incubation periods: first at 45 °C during 15 min, and after at room temperature during 30 min. Absorbance was recorded at 765 nm. The total phenolics content was calculated from a calibration curve prepared with gallic acid (linearity range: 5–100 mg/L; R^2^ = 0.9998) and expressed as µg of gallic acid equivalents (GAE) per mL of infusion. The assay was carried out in triplicate.

#### 2.4.5. Total Flavonoids Content

The total content of flavonoids present in the samples was determined as previously described [22]. Briefly, a sample aliquot (1 mL) was mixed with deionized water (4 mL) and 5% NaNO_2_ (300 µL), and the mixture was left to react for 5 min, at room temperature. Afterwards, 10% AlCl_3_ (300 µL) were added, followed by 1 M NaOH (2 mL) and distilled water (2.4 mL), 1 min later. Absorbance was measured at 510 nm. The total flavonoids content was calculated from a calibration curve prepared with catechin (linearity range: 2.5–400 µM; R^2^ = 0.9991) and expressed as µg of catechin equivalents (CE) per mL of infusion. The experiment was executed in triplicate. 

### 2.5. Analysis of Phenolic Compounds by UHPLC-ESI-QTOF-MS

The phenolic composition of the different infusions herein studied was analyzed exactly as described by Peixoto et al. [23]. Briefly, after lyophilization of the infusions, subsequent redissolution in 1 mL of methanol:water (1:1), and filtration throughout 0.22 μm nylon filter, the obtained extracts were analyzed in an UHPLC system (Agilent 1290, CA, USA) coupled to a Q-TOF mass spectrometer (Agilent 6540) equipped with an orthogonal ESI source. The chromatographic separation was performed at 40 °C using a Zorbax Eclipse Plus C18 column (2.1 × 10 mm, 1.8 μm; Agilent Technologies), with a gradient elution program at a flow rate of 0.5 mL/min and a sample injection volume of 2 μL. The eluents mixture was composed of H_2_O + HCOOH 0.01% (*v*/*v)* (eluent A) and ACN + HCOOH 0.01% (*v*/*v)* (eluent B) and was used as follows: 7′, 30% B; 9′, 80% B; 11′, 100% B; 13′, 100% B; 14′, 0% B. The MS and MS/MS spectra were obtained operating the mass spectrometer in the ESI negative ion mode (Agilent Jet Stream, AJS, Santa Clara, CA, USA). The tune method for MS analysis was as follows: capillary voltage, 3000 V; nebulizer pressure of 40 psi; drying gas flow rate, 8 L/min; gas temperature, 300 °C; skimmer voltage, 45 V; fragmentor voltage, 110 V. The MS and auto MS/MS modes were set to acquire *m*/*z* values ranging between 50–1100 and 50–800, respectively, at a scan rate of 5 spectra per second. The phenolic compounds were then tentatively identified using the Agilent Mass Hunter Qualitative analysis software (version B.07.00) and employing accurate mass data, MS/MS fragmentation patterns, ion source fragmentation, MS databases, and bibliographic research. Compounds’ identification was confirmed by reference standards when available. These standards were also useful to support the tentative annotation of structurally related compounds. Quantitative analysis was achieved using the Agilent Mass Hunter Quantitative analysis software for Q-TOF (version B.08.00). Compounds were quantified using reference standards whenever available, whereas tentatively annotated compounds were semi-quantified using reference standards with structural similarity. The instrumental linearity was tested using standards prepared in mobile phase and including ten concentration levels between 1 ng/mL and 1000 ng/mL. The calibration curves were obtained injecting each concentration level in duplicate. The response function was found to be linear for the target standards, which exhibited different linear ranges with determination coefficients (R^2^) between 0.9923–0.9998 (Table 1). LODs were calculated as the concentration giving a signal to noise ratio of three (S/N = 3). These limits were evaluated injecting standard solutions of the lowest concentration levels prepared in the mobile phase. Values ranging from 0.6 to 15 ng/mL were obtained. The high selectivity of LC-QTOF-MS/MS analysis allows for the dilution of the sample extracts prior, and matrix effects into the ion source was negligible. Intra (*n* = 3) and inter-day (*n* = 3) precisions were below 10%.

In order to avoid bias during the analysis, a randomized sequence of samples, including the replicates of each brand, was injected. For each sample, injections were made in triplicate. To monitor the stability of the analysis, a pool of all samples (containing equal aliquots from each one) was regularly injected through the samples sequence as quality control. In addition, in order to validate the UHPLC-HRMS method regarding retention time, signal variability, and mass accuracy, a second quality control, as well as a mix solution of ten standards, were also injected regularly throughout the samples’ sequence. 

### 2.6. Analysis of Free Amino Acids by RP-HPLC-FLD

Chromatographic analysis was performed in an integrated system from Jasco (Tokyo, Japan) equipped with a LC-NetII/ADC hardware interface, two pumps (PU-980), a high-performance autosampler (AS-4150), a column oven (CO-4061), and a fluorescence detector (FP-2020). The free amino acid profile of the different rosemary infusions was analyzed through automatic pre-column on-line derivatization (with OPA/3-MPA and FMOC) and RP-HPLC-FLD, as previously described [25]. Briefly, 1.5 mL of samples were first centrifuged at 13,000 rpm during 10 min (Heraeus Fresco 17 centrifuge, Thermo Fisher Scientific, Bremen, Germany). Afterwards, 990 µL of a centrifuged sample were mixed with 10 µL of norvaline (2 mg/mL, internal standard) and automatically derivatized. The procedure was performed in triplicate. A ZORBAX Eclipse Plus C18 (4.6 × 250 mm, 5 µm) column from Agilent Technologies (CA, USA) was used for compounds separation at 40 °C, using a gradient solvent system at a flow rate of 1.5 mL/min, which consisted of two mobile phases: (A) 10 mM Na_2_HPO_4_:10 mM Na_2_B_4_O_7_·10H_2_O:5 mM NaN_3_ (pH = 8.2); (B) MeOH:ACN:H_2_O (45:45:10, *v*/*v*/*v*). The gradient was as follows: 0.85′, 2% B; 33.4′, 57% B; 33.5′, 85% B; 39.3′, 85% B; 40.0′, 2% B. The volume of derivatized sample injected was 3 µL. Fluorescence detection was monitored for OPA-derivatives at λ_exc_ = 340 nm/λ_em_ = 450 nm (0.0–26.2 min), and for FMOC-derivatives at λ_exc_ = 266 nm/λ_em_ = 305 nm (26.2–40.0 min). Data were analyzed with the JASCO-ChromNAV software (version 2.02.08, Jasco, Tokyo, Japan). The compounds were identified by comparison with respective derivatized standards and quantified based on the internal standard method. The analytical performance was validated in terms of linearity (0.08–30 µg/mL for glycine, 0.6–60 µg/mL for lysine, and 0.15–240 µg/mL for the remaining amino acids, *r* ≥ 0.9995), intra-day (<3%) and inter-day precision (<8%) and limits of detection (0.100 µg/mL for lysine and 0.014–0.033 µg/mL for the remaining amino acids). The analysis was performed in triplicate, and the results were expressed in ng of amino acid per mL of infusion (ng/mL infusion). 

### 2.7. Statistical Analysis

Statistics was performed using the SPSS Statistics 26 for Windows program (IBM Corp., Armonk, NY, USA). One-Way ANOVA was firstly used to differences between samples, followed by Tukey’s HSD post-hoc test for pairwise comparisons between means. The results were considered statistically significant for *p* < 0.05. Linear relationships between different parameters was evaluated by Pearson correlation tests.

## 3. Results and Discussion

### 3.1. Antioxidant Activity and Total Contents in Phenolics and Flavonoids

The results obtained for the antioxidant activity and total phenolic contents of the different rosemary infusions studied are presented in Table 2. The phenolic contents are in accordance with those described in previous studies on rosemary [26,27,28]. However, rosemary infusions seem to possess lower contents of phenolic compounds and lower antioxidant potential than some herbal infusions commonly consumed worldwide, such as green tea, chamomile, sage, lemon balm, thyme, lemon grass, and echinacea infusions [26,27].

Comparing the results between samples, infusions from brand A presented the highest antioxidant activity in the FRAP assay, followed nearly by brands B, C, and D infusions. A similar trend was observed for total phenolics and flavonoids: brand A infusions presented the highest contents of phenolics, whereas brand D showed the lowest contents. In the ORAC assay, the results were partially similar to those obtained for the abovementioned assays, with brand A infusions presenting significantly higher results, while brand C ones showed the lowest values. However, in the DPPH^•^ scavenging assay, the results showed a reversed trend, with brand D infusions exhibiting the highest radical scavenging ability. The observed differences between the samples can be related to the different content in phenolics and to the existence of other compounds with antioxidant potential, as further discussed in Section 3.3.

### 3.2. Phenolic Profiling by UHPLC-ESI-QTOF-MS

In order to estimate the influence of the chemical composition on the antioxidant activity of rosemary infusions, a comprehensive phenolic profiling analysis was carried out based on the information provided by high-resolution mass spectrometry (HRMS/MS) data, MS databases search (e.g., Metlin and HMDB) and data described in literature. When available, reference standards were used to confirm the identification of compounds by comparing their retention times and HRMS/MS data, also supporting the tentative identification of some structurally related compounds. 

A total of forty-four phenolic compounds, belonging to nine different groups, were tentatively identified in rosemary infusions: seven hydroxybenzoic acids (**1**–**4**, **6**, **7**, and **12**), seven hydroxycinnamic acids (**9**–**11**, **13**, **16**, **18**, and **24**), one flavan-3-ol (**15**), one flavanone (**25**), thirteen flavones (**17**, **19**–**23**, **26**–**30**, **34**, and **37**), eleven phenolic diterpenes (**31**–**33**, **35**, **36**, **39–44**), one hydroxybenzaldehyde (**5**), two coumarins (**8** and **14**) and one pyranochromanone (**38**). Table 3 summarizes the identified compounds, as well as their chromatographic characteristics and HRMS data. 

**Figure 1:**
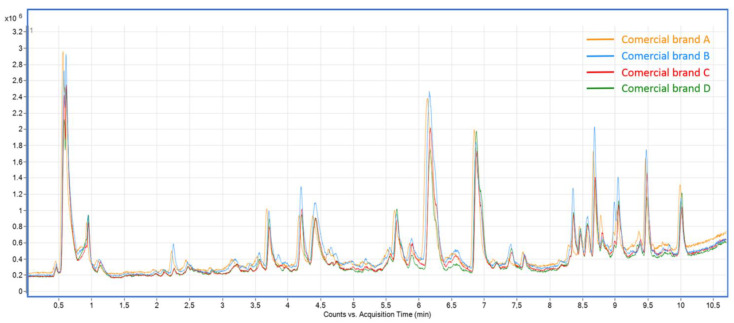
Total ion chromatograms (TIC) of rosemary infusions from different brands, analyzed by UHPLC-ESI-QTOF-MS/MS.

Table 4 presents a quantitative approach of the tentatively identified compounds. As previously mentioned, whenever available, the identified compounds were quantified using reference standards, whereas tentatively annotated compounds were semi-quantified using reference standards with structural similarity. The results are expressed as ng of compound/mL of infusion. Table 4 also contains the accumulated amounts of the different classes and subclasses of compounds in rosemary infusions, which was quite similar between all commercial brands: phenolic diterpenes constituted the main group of compounds, followed by hydroxycinnamic acids and flavan-3-ols groups. A color code ranging from high concentration (dark color) to low concentration (light color) allow us to follow the amount of each compound in the analyzed infusions. In addition, Figure 1 presents the total ion chromatograms (TIC) of the different infusions, where the similarities between them can be noticed.

Considering the chemical characterization of the compounds detected in rosemary infusions, their tentative identification will be discussed by classes or subclasses. In relation to hydroxybenzoic acids, most of the compounds identified are structurally related with each other: compounds **1** and **6** were tentatively identified as hexosides of compounds **4** (protocatechuic acid) and **7** (4-hydroxybenzoic acid), respectively, because of the loss of a hexose moiety (162 Da) which generated fragments ions at *m*/*z* 153 and 137, and which correspond to their respective aglycones. Moreover, the typical fragments at *m*/*z* 109 and 93, also detected in compounds **4** and **7**, respectively, and originated due to loss of CO_2_ from the respective precursor ions (153 and 137) also helped to better elucidate the identification of compounds **1** and **6**. As far as we know, compound **1** (a dihydroxybenzoic acid hexoside) is being identified for the first time in rosemary infusions. Similarly to compounds **4** and **7**, compound **3** (hydroxymethoxybenzoic acid) also loses a CO_2_ moiety (44 Da) from the corresponding precursor ion, characteristic of hydroxybenzoic acid derivatives [20]. Moreover, it also presents a fragment ion at *m*/*z* 108, which is commonly found in MS/MS databases of hydroxymethoxybenzoic acid isomers, and which was also detected for compound **12**, another hydroxymethoxybenzoic acid isomer. Compound **2** (hydroxydimethoxybenzoic acid), unlike others, generate a base peak at *m*/*z* 135 through the successive loss of a water molecule and a CO_2_ molecule ([M − H − H_2_O − CO_2_]^−^) and a *m*/*z* 123 [M − H − C_3_H_6_O_2_]^−^ fragment, which seems to result from the loss of a methoxyethenol group. 

The only hydroxybenzaldehyde derivative found in rosemary infusions (compound **5**) was identified as dihydroxybenzaldehyde. The pattern of fragmentation was quite similar to MS spectra available on databases, which facilitated the identification of the compound. The more relevant fragment ions at *m*/*z* 108 and 119 resulted, from the loss of the aldehyde moiety and of a water molecule, respectively. As far as we know, this is the first time that dihydroxybenzaldehyde is being described for rosemary infusions. 

Regarding the coumarins class, two compounds with the same deprotonated molecular ions were identified as dihydroxycoumarin hexoside I (compound **8**) and dihydroxycoumarin hexoside II (compound **14**). These compounds, apparently detected for the first time in rosemary infusions (only in brand C), presented the same base peak at *m*/*z* 177 as a result of the neutral loss of a hexose moiety (162). Further characterization of these compounds is required, as they may correspond to various dihydroxycoumarin hexosides, such as esculin, cichoriin, or daphnin, already found in other plants and with reported biological properties [29,30,31,32,33,34]. For example, esculin has been proved to inhibit lipid peroxidation and to scavenge hydroxyradicals, as well as to possess anti-inflammatory activity and gastroprotective effects [30,33]. Interestingly, all of these properties are also described for rosemary and, to some extent, might be associated with these compounds (even though they have been found in quite low quantities).

As observed for hydroxybenzoic acids, identified hydroxycinnamic acids are structurally related to each other. For compounds **9** and **10**, the loss of a hexose moiety (162 Da) leads to fragment ions at *m*/*z* 179 and 163, together with their correspondingly characteristic fragment ions at *m*/*z* 135 and 119, both resulted from the loss of a CO_2_ moiety, which allowed their identification as hexosides of the compounds **13** (caffeic acid) and **16** (*p-*coumaric acid). As far as we know, coumaric acid hexoside (compound **10**) was detected for the first time in rosemary. Compound **11** was identified as caffeoylquinic acid according to its characteristic fragments at *m*/*z* 191 (quinic acid), *m*/*z* 179 (caffeic acid), and the characteristic *m*/*z* 173 (loss of a water from quinic acid) of 4-caffeoylquinic acid (cryptochlorogenic acid) where this fragment represents the base peak, and its identity was confirmed by its reference standard. The most relevant hydroxycinnamic acid found in rosemary infusions was rosmarinic acid (compound **24**). This compound, which presented the highest concentrations in all infusions representing around 28–31% of the total compounds detected, is well described in literature as a major compound of the plant [1,2,5,9] and, in fact, is considered a “family marker” for the Lamiaceae family [35]. It is noteworthy that rosmarinic acid is endowed by numerous beneficial properties, such as antioxidant, antiulcerogenic, antidepressant, and anti-inflammatory properties and, therefore, is considered one of the main responsible for the effects reported for rosemary infusions [3,4,5,8]. Concerning its fragmentation pattern, as a caffeic acid derivative, rosmarinic acid presented, as described, a typical fragment ion at *m*/*z* 179 corresponding to caffeic acid, as well as two caffeic acid fragments at *m*/*z* 161 and 135, resulted from the loss of a water molecule and a CO_2_ moiety, respectively. The fragment ion found at *m*/*z* 197 is also characteristic of rosmarinic acid and corresponds to the 2-hydroxy derivative of hydrocaffeic acid [20]. Additionally, a hexoside of rosmarinic acid was also detected in rosemary (compound 18), showing the characteristic fragments of rosmarinic acid and [M − H − C_6_H_10_O_5_]^−^ and [M − H − C_9_H_10_O_5_]^−^ ions—resultant from the loss of the hexose and the 2-hydroxy-hydrocaffeic acid moieties, respectively—in the MS/MS spectra.

Flavonoids represented one of the main groups of compounds found in rosemary infusions, although the results were a bit dissimilar between the three identified flavonoid subclasses. Despite being the only flavan-3-ol identified in the studied infusions, compound **15** represents around 7–10% of the total compounds detected, making the flavan-3-ol subclass the main flavonoid subclass detected. The MS/MS data of compound **15** showed fragment ions at *m*/*z* 97 and 225, which are well documented in literature and databases as gallocatechin. Gallocatechin is well described as one of the major flavonoids found in rosemary extracts [2,9,20].

Flavones’ subclass includes a total of thirteen compounds identified at relatively low abundance, accounting for about 3–6% of the total compounds detected in the different rosemary infusions. Compounds **17**, **21**, **22**, and **26** were identified as hexosides of hydroxyluteolin/quercetin, luteolin, nepetin and hispidulin, respectively, because of the neutral loss of a hexose moiety (162 Da), which resulted in the respective aglycone. For compound **17**, two different flavonoids (the flavone hydroxyluteolin-*O*-glucoside and the flavonol quercetin-*O*-glucoside) are documented to be present in rosemary [2,5,20]. Although it was not possible to completely establish the identification of compound **17** due to the lack of standards, this compound might correspond to an hexoside of the flavone hydroxyluteolin, as this compound did not contain a fragment ion at *m*/*z* 300, which is usually found for quercetin derivatives. The unambiguous identification of compound **21** was confirmed by comparing its retention time and MS/MS data with its corresponding reference standard. Compounds **19** and **23** were identified as rutinosides of luteolin and apigenin, as deducted from the neutral loss of 308 Da and the resulting fragment ions at *m*/*z* 285 and 269, respectively. Compound **19** also exhibited a characteristic fragment ion of luteolin, at *m*/*z* 151, that helped to confirm the identity of the compound.

Like hexosides and rutinosides, glucuronide compounds can be readily identified based on the neutral loss of the glucuronide moiety (176 Da), which results in the corresponding aglycones ([M − H − 176]^−^). Thus, compounds **20** and **27** detected in rosemary infusions were identified as glucuronides of two isomers (luteolin and scutellarein), both already described for rosemary. Compounds **28**, **29**, and **30** were identified as three tetrahydroxyflavone-*O-*acetylglucuronide isomers based on the neutral loss of an acetylglucuronide moiety (218 Da), leading to a fragment ion at *m*/*z* 285, corresponding to various possible tetrahydroxyflavone (e.g., luteolin and scutelarrein). These compounds could be assigned as luteolin-*O-*acetylglucuronide isomers, based on data reported in the literature [2,5,9]. More specifically, according to Borras-Linares et al. (2014), compounds **28** and **29** could be identified as luteolin-3′-*O-(*2″-*O-*acetyl)-β-d-glucuronide isomers I and II based on the presence of fragment ions at *m*/*z* 285 and 399 which correspond to [M − H − C_8_H_10_O_7_]^−^ and [M − H − C_3_H_4_O_4_]^−^, whereas compound 30 could be identified as luteolin-3′-*O-(*3″-*O-*acetyl)-β-d-glucuronide or as luteolin-3′-*O-(*4″-*O-*acetyl)-β-d-glucuronide based on fragment ions at *m*/*z* 285 and 443, which are common to both isomers, and corresponding to acetylglucuronide and acetyl moieties, respectively [2]. Notwithstanding, their unambiguous identity cannot be confirmed by the analytical method employed in this work, and this is why they were generically identified as tetrahydroxyflavone-*O-*acetylglucuronide isomers. In turn, compounds **34** and **37** were identified as methoxyflavones’ derivatives, due to the characteristic neutral loss of methyl groups (15 Da), and, according to data reported for rosemary [2,5,9,20], they were assigned as cirsimaritin and genkwanin. Finally, compound **25**, the only flavanone identified, was assigned as hesperidin, as deducted from the loss of a rutinoside moiety, as well as based on data found in the literature search [2,5,9], and its identity was then confirmed with the reference standard of the compound.

The phenolic diterpenes identified in this work represent the most abundant group detected in these infusions, in accordance with other studies about rosemary phytochemical composition [2,9,21]. A total of eleven phenolic diterpenes have been detected, two of which (compound **33** and **41**) stood out particularly due to their high concentration. Compound **33**, together with compounds **31** and **35**, were identified as (epi)(iso)rosmanol. These three isomeric forms (rosmanol, epirosmanol and episorosmanol) are equally described in the literature for rosemary [2,5,9] and cannot be distinguished by the analytical method employed in this work, exhibiting characteristic fragment ions at *m*/*z* 301 and 283 resulted, respectively, from the loss of a CO_2_ molecule ([M − H − CO_2_]^−^), followed by the loss of a water molecule ([M − H − CO_2_ − H_2_O]^−^). Similar neutral losses were found for compound **32**, a rosmanol derivative that shows the base peak at *m*/*z* 359—an *m*/*z* equal to the deprotonated molecular ion of compound **40**, which was previously described by Borrás-Linares et al. (2014) as epirosmanol methyl ether [2]. Compound **41** (carnosol), whose presence in rosemary is already well documented in several studies, show the characteristic base peak detected at *m*/*z* 285, which results from the loss of a CO_2_ molecule ([M − H − CO_2_]^−^). The same loss was also observed for compound **44**, which was identified as carnosic acid, as well as for its isomer (compound **36**). This phenolic diterpene is highly reported as the strongest antioxidant compound and one of the main compounds in rosemary [1,2,5,9,20,21], although it was found in the studied infusions at very low levels. This can be explained due to its instability in the presence of oxygen and heat, leading to degradation into other phenolic diterpenes, such as carnosol, rosmanol, epirosmanol, epiisorosmanol, and rosmadial, which were found in high amounts in the studied samples [9]. Compounds **39** and **42** presented the same deprotonated molecular ions and might correspond to different isomeric forms already described in the literature [2,9,20]. The fragment ions found in each compound at *m*/*z* 299 and 315 from losses of propyl and ethylene moieties, respectively, are typical fragments of rosmadial and safficinolide. Compound **43**, characterized by the loss of a methyl group from the precursor ion, was identified as rosmaridiphenol as reported in literature and databases [2,9].

Finally, a compound that does not belong to any of the compound families discussed above, was identified as isocalolongic acid (compound **38**)—a pyranochromanone with reported bioactive potential, previously identified in plants of the genus *Calophyllum* [36,37]—based on the positive match of MS/MS data in MS databases. To the best of our knowledge, this compound in rosemary is being described for the first time. 

### 3.3. Free Amino Acid Profile by RP-HPLC-FLD

The free amino acid content obtained by RP-HPLC-FLD analysis of the different rosemary infusions is shown in Table 5. Seven proteinogenic amino acids were detected (asparagine, threonine, alanine, tyrosine, phenylalanine, isoleucine, and proline), and four of them were found in all commercial brands (alanine, phenylalanine, isoleucine, and proline). Brand C infusions presented the highest content of total free amino acids (presenting the seven amino acids detected), whereas brand A showed the lowest ones (presenting only the four amino acids common to all brands). Although rosemary infusions seem to be poor in free amino acids, it is important to note that, even in small quantities, its consumption may help to maintain the physiological levels of these amino acids in the organism and the corresponding homeostasis [16,38]. In particular, these small amounts might have contributed to the antioxidant properties of rosemary infusions, even because some of the amino acids present have their antioxidant activity well described in the literature. For example, Guidea et al. (2020) found that asparagine presented the highest capacity to scavenge superoxide radicals and to chelate metal ions, compared to the other amino acids [15]. In the same study, phenylalanine and isoleucine (both present in all the samples) showed significant abilities to scavenge DPPH^•^ and superoxide radicals, but relatively low reducing power in FRAP assay [15]. In another piece of research, the amino acids with hydrophobic residues, such as alanine, isoleucine, phenylalanine, and proline (all detected in the samples), protected myoglobin from oxidation by peroxyl radicals [39]. Thus, these studies reveal the antioxidant potential of some amino acids and show that, depending on their side chain residues, they can exert their antioxidant activity by different mechanisms of actions (scavenging of different radicals, reducing power, and/or chelating capacity), to a greater or lesser degree [15,39].

### 3.4. Impact of Phenolics and Free Amino Acids on the Antioxidant Potential

In order to estimate the impact of phenolics in the antioxidant activity of rosemary infusions, a Pearson correlation analysis between the total phenolics and flavonoids contents and DPPH^•^ scavenging, FRAP, and ORAC assays was performed (Table 6). High correlations were found between the total phenolics and flavonoids and FRAP values, suggesting that phenolics (including the flavonoid class) of rosemary infusions exert antioxidant activity preferentially via electron transfer mechanisms. Although rosemary infusions also presented high capacity to inhibit peroxyl radicals by H atom transfer mechanisms in the ORAC assay, no significant correlations were detected between the total contents of phenolics and flavonoids and ORAC values. Therefore, it can be assumed that other antioxidant compounds present in these infusions beyond phenolics might be able to prevent lipid peroxidation in biological systems. In fact, the results obtained for DPPH^•^ scavenging assay also suggest the presence of other type of antioxidant compounds, since negative correlations were found between DPPH^•^ assay and total contents of phenolics and flavonoids. In addition, negative correlations were also found between DPPH^•^ scavenging assay and FRAP and ORAC assays, which can be explained based on the mechanisms of action implicated in each of these methods. While in the DPPH^•^ scavenging assay the radical may be neutralized by radical quenching via H atom transfer or by direct reduction via electron transfer, in the FRAP method, the mechanism of the reaction is mainly via electron transfer (the Fe^3+^-TPTZ complex being reduced to Fe^2+^-TPTZ by compounds with a redox potential <0.7 V), whereas in the ORAC assay is mainly via H atom transfer (where antioxidants present in the sample transfer H atoms to peroxyl radicals and protect fluorescein from degradation by these radicals) [40,41]. Thus, while antioxidant activity evaluated by FRAP and ORAC assays seems to be mainly due to the presence of phenolics, DPPH^•^ scavenging capacity cannot be solely attributed to phenolics but also to other antioxidants.

In fact, compounds such as abietane diterpenes, triterpenes and volatile compounds (e.g., 1,8-cineole, camphor, borneol, verbenone) are well documented in the literature for rosemary, as well as their respective antioxidant properties [2,3,9,10,21,42]. More specifically, the abietane diterpenes containing two hydroxyl groups in C_11_ and C_12_, such as carnosol, rosmanol, carnosic acid, and other oxidized diterpenes were mainly responsible for the free radical scavenging activity in rosemary extracts [21]. As noted in Table 4, these compounds were present in high amounts in rosemary infusions, representing the main group of compounds found. According to this, it would be expected that the antiradical activity (DPPH^•^) of rosemary infusions from both commercial brands C and D was higher than the other commercial brands, due to the higher concentrations of phenolic diterpenes in these samples. However, it is worth highlighting possible interactions between the identified bioactive compounds. For example, according to Almela et al. [21], flavones interact with phenolic diterpenes in a synergistic manner. Thus, the higher accumulated concentration of flavones in commercial brand D compared to brand C might enhance the synergistic effect between phenolic diterpenes and flavones, which might explain the stronger DPPH^•^ scavenging potential of brand D infusion. Moreover, it is important to highlight that, in the DPPH^•^ scavenging assay, both electron and H atom transfers are involved, so it is understandable that, in complex matrices such as plant infusions—in which the compounds are present in different proportions and may exert its antioxidant activity by different mechanisms of action—interactions (e.g., synergisms and antagonisms) may occur between compounds which can lead to quite variable and unexpectable results in this assay. 

Besides phenolic compounds and the other compounds above mentioned, bearing in mind the data reported in literature about the antioxidant potential of amino acids and their derivatives, it is possible that the free amino acids detected in rosemary infusions also had an impact on their antioxidant properties. Notwithstanding, it is important to note that not all amino acids are endowed of antioxidant properties, and, besides the mechanisms of action previously mentioned in Section 3.3, amino acids can also act by others—namely by enhancing the activity of antioxidant enzymes or by acting as oxidative stress biomarkers [15,43]. Nonetheless, considering the low concentration levels of amino acids (low ng/mL) compared to the high concentration levels of phenolic compounds (high ng/mL to low μg/mL), it is evident that phenolic compounds (including flavonoids and phenolic diterpenes) were the principal responsible for the antioxidant potential of rosemary infusions.

Overall, it can be said that rosemary infusions are a good source of antioxidant compounds: a cup of 200 mL of infusion—which is the amount normally prepared and ingested by consumers according to manufacturers’ recommendations—may contain around 30 mg of phenolic compounds and a mean value of 800 ng of free amino acids. In this way, it is possible that rosemary infusions may present a positive impact on the prevention of oxidative stress related diseases. The antioxidant experiments performed in this study, although very useful, use synthetic free radicals or metal ions to evaluate the ability of these infusions in neutralizing or reducing the oxidative stress generated by them, which have some limitations. Therefore, it would be important in the future (in order to understand if the obtained results can be extended to an in vivo system), to perform in vitro simulation of gastrointestinal digestion and cell assays (using, for example, a Transwell system with Caco-2 cells) to account with the bioacessibility and bioavailability issues that result from gastrointestinal digestion. By that method, it will also be possible to evaluate the effect of the infusion directly on Caco-2 cells and the effect of the permeate in other cell lines. This would give us a more realistic approach of what could happen in a real situation.

## 4. Conclusions

This study allowed us to perceive the impact of phenolics, flavonoids, and free amino acids on the antioxidant activity of rosemary infusions and also the richness of these infusions in these antioxidant compounds. As expected, phenolic compounds proved to be the main responsible for the antioxidant activity of rosemary infusions, seeming to act preferentially by electron transfer mechanisms. In addition, the slight differences observed in the chemical composition of the different commercial brands might have resulted in remarkable synergisms and/or antagonisms between compounds, and, consequently, in different antioxidant activities. Thus, in further studies, it would be interesting to study these possible interactions, including interactions between phenolics and free amino acids (which, although herein detected in minor amounts, are also known to possess antioxidant properties), and also the antioxidant potential of rosemary infusions in a biological system that could mimic an in vivo situation.

## Figures and Tables

**Table 1:** Linearity parameters and limits of detection (LOD) of phenolic standards used for quantification.

Compounds	Linearity		LOD (ng/mL)
	R^2^	Range (ng/mL)	
Protocatechuic acid	0.9976	10–1000	3.0
Syringic acid	0.9994	10–1000	3.0
Vanillic acid	0.9988	10–1000	3.0
4-hydroxybenzoic acid	0.9944	2–50	0.6
p-Coumaric acid	0.9985	2–100	0.6
Caffeic acid	0.9996	2–1000	0.6
Chlorogenic acid	0.9995	10–1000	3.0
Catechin	0.9993	5–1000	1.5
Luteolin-7-*O*-glucoside	0.9943	10–1000	3.0
Rosmarinic acid	0.9981	20–1000	6.0
Rutin	0.9975	5–1000	1.5
Hesperidin	0.9986	5–1000	1.5
Carnosol	0.9995	2–50	0.6
Hesperetin	0.9923	2–1000	0.6
Carnosic acid	0.9987	50–1000	15.0
Genkwanin	0.9998	2–50	0.6

**Table 2:** Antioxidant activity, total phenolics and flavonoids of rosemary infusions from different brands.

Commercial Brand	Antioxidant Activity			Bioactive Compounds	
	DPPH Inhibition (μg TE/mL)	FRAP (μg FSE/mL)	ORAC (μg TE/mL)	Total Phenolic Content (μg GAE/mL)	Total Flavonoids Content (μg CE/mL)
A	16.40 ± 0.47 ^b^	729.67 ± 55.93 ^a^	157.51 ± 14.61 ^a^	34.65 ± 1.71 ^a^	31.22 ± 1.09 ^a^
B	12.98 ± 1.53 ^b^	564.67 ± 55.08 ^b^	128.17 ± 12.47 ^b^	27.65 ± 1.27 ^b^	26.64 ± 0.52 ^b^
C	16.65 ± 1.06 ^b^	514.67 ± 53.93 ^b^	91.72 ± 5.75 ^c^	26.35 ± 1.23 ^bc^	24.69 ± 1.93 ^bc^
D	22.82 ± 1.77 ^a^	451.33 ± 17.56 ^b^	126.47 ± 11.28 ^b^	23.06 ± 0.59 ^c^	21.36 ± 1.29 ^c^

The results are expressed as mean ± standard deviation. Within each column, different letters denote significant differences between mean values (*p* < 0.05). DPPH^•^, 1,1-diphenyl-2-picrylhydrazyl radical; FRAP, Ferric reducing antioxidant power; ORAC, Oxygen radical absorbance capacity; TE, Trolox equivalent; FSE, ferrous sulfate equivalents; GAE, gallic acid equivalents; CE, catechin equivalents.

**Table 3:** Tentative identification of compounds detected in rosemary infusions by UHPLC-ESI-QTOF-MS.

Peak	Retention Time (min)	[M − H]^−^ Experimental	[M − H]^−^ Theorical	Error (ppm)	MS^2^ Product Ions	Molecular Formula	Family	Tentative Identification	Std **	Ref.
1	2.144	315.0717	315.0722	1.46	108 (42), 109 (38), 152 (100), 153 (41)	C_13_H_16_O_9_	Hydroxybenzoic acid	Dihydroxybenzoic acid hexoside	A	–
2	2.260	197.0454	197.0455	0.75	123 (75), 135 (100)	C_9_H_10_O_5_	Hydroxybenzoic acid	Hydroxydimethoxybenzoic acid	B	[2,5,20]
3	2.334	167.0344	167.0350	3.49	108 (81), 121 (100), 123 (82), 137 (76)	C_8_H_8_O_4_	Hydroxybenzoic acid	Hydroxymethoxybenzoic acid (I)	C	[20]
4	2.467	153.0195	153.0193	−1.09	109 (100)	C_7_H_6_O_4_	Hydroxybenzoic acid	Protocatechuic acid *	A	[20]
5	3.141	137.0239	137.0244	3.82	108 (48), 119 (9), 136 (53), 137 (100)	C_7_H_6_O_3_	Hydroxybenzaldehyde	Dihydroxybenzaldehyde	A	–
6	3.181	299.0777	299.0772	−1.53	93 (100), 137 (99)	C_13_H_16_O_8_	Hydroxybenzoic acid	Hydroxybenzoic acid-*O*-hexoside	D	[20]
7	3.221	137.0246	137.0244	−1.32	93 (37)	C_7_H_6_O_3_	Hydroxybenzoic acid	4-hydroxybenzoic acid *	D	[20]
8	3.411	339.0719	339.0722	0.76	177 (100), 221 (10)	C_15_H_16_O_9_	Coumarin	Dihydroxycoumarin hexoside (I)	E	–
9	3.567	341.0876	341.0878	0.66	135 (61), 161 (20), 179 (100), 221 (25), 281 (30)	C_15_H_18_O_9_	Hydroxycinnamic acid	Caffeic acid-*O-*hexoside	F	[20]
10	3.574	325.0926	325.0929	1.00	119 (49), 163 (100)	C_15_H_18_O_8_	Hydroxycinnamic acid	Coumaric acid hexoside	E	–
11	3.667	353.0878	353.0878	0.02	173 (44), 179 (69), 191 (100)	C_16_H_18_O_9_	Hydroxycinnamic acid	Caffeoylquinic acid *	G	[5,20]
12	3.811	167.0350	167.0350	−0.10	108 (100), 152 (55)	C_8_H_8_O_4_	Hydroxybenzoic acid	Hydroxymethoxybenzoic acid (II)	C	[20]
13	3.874	179.0351	179.0350	−0.65	135 (100)	C_9_H_8_O_4_	Hydroxycinnamic acid	Caffeic acid *	F	[1,5,20]
14	3.907	339.0724	339.0722	−0.71	177 (100)	C_15_H_16_O_9_	Coumarin	Dihydroxycoumarin hexoside (II)	E	–
15	4.427	305.0706	305.0667	−12.85	59 (43), 97 (100), 225 (40)	C_15_H_14_O_7_	Flavan-3-ol	Gallocatechin	H	[2,9,20]
16	4.747	163.0398	163.0400	1.37	119 (100)	C_9_H_8_O_3_	Hydroxycinnamic acid	*p-*Coumaric acid *	E	[20]
17	5.077	463.0877	463.0882	1.09	301 (100)	C_21_H_20_O_12_	Flavone/Flavonol	Hydroxyluteolin/Quercetin-*O*-hexoside	I	[2,5,20]
18	5.371	521.1293	521.1301	1.47	135 (19), 161 (33), 179 (59), 197 (42), 323 (86), 359 (51)	C_24_H_26_O_13_	Hydroxycinnamic acid	Rosmarinic acid-*O*-hexoside	J	[2,5]
19	5.521	593.1517	593.1512	−0.85	285 (65), 151 (22), 593 (100)	C_27_H_30_O_15_	Flavone	Luteolin-*O*-rutinoside	K	[2,5,9,20]
20	5.567	461.0726	461.0726	−0.10	285 (100)	C_21_H_18_O_12_	Flavone	Luteolin/Scutellarein-*O*-glucuronide (I)	I	[1,2,5,9,20]
21	5.641	447.0942	447.0933	−2.04	285 (100)	C_21_H_20_O_11_	Flavone	Luteolin-7-*O*-glucoside *	I	[20]
22	5.898	477.1046	477.1039	−1.56	299 (20), 315 (47), 477 (100)	C_22_H_22_O_12_	Flavone	Nepitrin	I	[2,5,9,20]
23	5.908	577.1561	577.1563	0.32	269 (100)	C_27_H_30_O_14_	Flavone	Apigenin-*O*-rutinoside	K	[20]
24	6.188	359.0775	359.0772	−0.71	135 (7), 161 (100), 179 (12), 197 (52)	C_18_H_16_O_8_	Hydroxycinnamic acid	Rosmarinic acid *	J	[1,2,5,9,20]
25	6.291	609.1825	609.1825	0.00	301 (100)	C_28_H_34_O_15_	Flavanone	Hesperidin *	L	[2,5,9]
26	6.454	461.1091	461.1089	−0.35	283 (32), 297 (10), 461 (100)	C_22_H_22_O_11_	Flavone	Homoplantaginin	I	[1,2,5,9]
27	6.568	461.0728	461.0726	−0.53	285 (100)	C_21_H_18_O_12_	Flavone	Luteolin/Scutellarein-*O*-glucuronide (II)	I	[1,2,5,9,20]
28	7.061	503.0840	503.0831	−1.75	285 (44), 399 (100)	C_23_H_20_O_13_	Flavone	Tetrahydroxyflavone-*O*-acetylglucuronide (I)	I	[1,2,5,9]
29	7.314	503.0833	503.0831	−0.36	285 (100), 399 (11)	C_23_H_20_O_13_	Flavone	Tetrahydroxyflavone-*O*-acetylglucuronide (II)	I	[1,2,5,9]
30	7.741	503.0828	503.0831	0.63	285 (100), 443 (14)	C_23_H_20_O_13_	Flavone	Tetrahydroxyflavone-*O*-acetylglucuronide (III)	I	[1,2,5,9]
31	8.365	345.1715	345.1707	−2.18	301 (100)	C_20_H_26_O_5_	Phenolic diterpene	(Epi)(iso)Rosmanol (I)	M	[2,5,9,20]
32	8.571	403.1765	403.1762	−0.68	281 (34), 299 (18), 341 (15), 359 (100)	C_22_H_28_O_7_	Phenolic diterpene	Rosmanol derivative	M	–
33	8.691	345.1719	345.1707	−3.34	283 (44), 301 (100)	C_20_H_26_O_5_	Phenolic diterpene	(Epi)(iso)Rosmanol (II)	M	[2,5,9,20]
34	8.718	313.0716	313.0718	0.52	283 (100), 298 (83)	C_17_H_14_O_6_	Flavone	Cirsimaritin	N	[2,5,20]
35	8.798	345.1711	345.1707	−1.02	283 (100), 301 (19)	C_20_H_26_O_5_	Phenolic diterpene	(Epi)(iso)Rosmanol (III)	M	[2,5,9,20]
36	8.848	331.1918	331.1915	−0.96	283 (26), 287 (12), 331 (100)	C_20_H_28_O_4_	Phenolic diterpene	Carnosic acid isomer	M	[2,5,9,20]
37	8.961	283.0610	283.0612	0.70	268 (100)	C_16_H_12_O_5_	Flavone	Genkwanin *	O	[2,5,9,20]
38	9.051	387.1819	387.1813	−1.49	179 (8), 283 (43), 343 (100)	C_22_H_28_O_6_	Pyranochromanone	Isocalolongic acid	M	–
39	9.345	343.1550	343.1551	0.29	299 (34), 343 (100)	C_20_H_24_O_5_	Phenolic diterpene	Rosmadial/Safficinolide (I)	M	[2,9,20]
40	9.445	359.1864	359.1864	0.00	283 (89), 284 (100), 285 (49), 300 (52), 317 (64)	C_21_H_28_O_5_	Phenolic diterpene	Epirosmanol methyl ether	M	[2]
41	9.488	329.1766	329.1758	−2.33	285 (100)	C_20_H_26_O_4_	Phenolic diterpene	Carnosol	M	[2,9,20]
42	9.591	343.1552	343.1551	−0.30	315 (55), 343 (100)	C_20_H_24_O_5_	Phenolic diterpene	Rosmadial/Safficinolide (II)	M	[2,9,20]
43	9.725	315.1957	315.1966	2.76	285 (100)	C_20_H_28_O_3_	Phenolic diterpene	Rosmaridiphenol	M	[2,9]
44	9.838	331.1912	331.1915	0.86	287 (100)	C_20_H_28_O_4_	Phenolic diterpene	Carnosic acid *	M	[2,5,9,20]

*, Confirmed by reference standard. **, Reference standard used for quantification or semi-quantification: A: Protocatechuic acid, B: Syringic acid, C: Vanillic acid, D: 4-hydroxybenzoic acid, E: *p*-Coumaric acid, F: Caffeic acid, G: Chlorogenic acid, H: Catechin, I: Luteolin-7-*O*-glucoside, J: Rosmarinic acid, K: Rutin, L: Hesperidin, M: Carnosic acid, N: Hesperetin, O: Genkwanin.

**Table 4:** Quantification of compounds detected in rosemary infusions by UHPLC-ESI-QTOF-MS (ng of compound/mL of infusion).

Peak	Compound	Std *	Concentration (ng/mL of Infusion)			
			A	B	C	D
1	Dihydroxybenzoic acid hexoside	A	343.3 ± 1.8 ^ab^	223.0 ± 23.5 ^b^	364.3 ± 64.9 ^ab^	456.1 ± 5.4 ^a^
2	Hydroxydimethoxybenzoic acid	B	516.4 ± 198.5 ^b^	1269.9 ± 32.4 ^a^	1190.9 ± 6.3 ^a^	1134.3 ± 35.0 ^a^
3	Hydroxymethoxybenzoic acid (I)	C	489.2 ± 82.6 ^a^	487.1 ± 89.8 ^a^	524.6 ± 79.5 ^a^	544.9 ± 47.5 ^a^
4	Protocatechuic acid	A	325.7 ± 46.8 ^a^	458.5 ± 91.5 ^a^	325.9 ± 54.8 ^a^	456.3 ± 36.3 ^a^
5	Dihydroxybenzaldehyde	A	1125.6 ± 37.2 ^a^	851.8 ± 88.9 ^a^	452.7 ± 76.4 ^b^	436.5 ± 12.7 ^b^
6	Hydroxybenzoic acid-*O*-hexoside	D	62.3 ± 1.2 ^a^	33.8 ± 5.3 ^a^	51.3 ± 8.3 ^a^	59.4 ± 2.3 ^a^
7	4-hydroxybenzoic acid	D	228.8 ± 6.4 ^a^	165.9 ± 18.4 ^a^	216.2 ± 50.5 ^a^	257.9 ± 7.1 ^a^
8	Dihydroxycoumarin glucoside (I)	E	n.d.	n.d.	29.7 ± 4.9	n.d.
9	Caffeic acid-*O-*hexoside	F	189.6 ± 52.6 ^a^	134.8 ± 14.3 ^a^	126.5 ± 10.2 ^a^	119.0 ± 9.9 ^a^
10	Coumaric acid hexoside	E	65.5 ± 0.6 ^a^	63.8 ± 4.0 ^a^	88.3 ± 10.1 ^a^	89.5 ± 6.9 ^a^
11	Caffeoylquinic acid	G	322.2 ± 81.5 ^a^	361.5 ± 79.7 ^a^	841.3 ± 52.2 ^a^	711.9 ± 148.6 ^a^
12	Hydroxymethoxybenzoic acid (II)	C	423.9 ± 32.8 ^a^	300.9 ± 15.0 ^a^	448.2 ± 85.4 ^a^	494.1 ± 24.4 ^a^
13	Caffeic acid	F	97.0 ± 23.0 ^b^	460.5 ± 93.1 ^a^	298.7 ± 6.8 ^ab^	144.4 ± 12.4 ^b^
14	Dihydroxycoumarin glucoside (II)	E	n.d.	n.d.	60.6 ± 36.6	n.d.
15	Gallocatechin	H	15,352.1 ± 831.4 ^a^	10,574.4 ± 1057.5 ^a^	13,920.5 ± 1968.0 ^a^	16,253.0 ± 577.3 ^a^
16	*p-*Coumaric acid	E	157.1 ± 9.0 ^ab^	151.4 ± 8.6 ^b^	125.6 ± 3.0 ^b^	187.3 ± 5.4 ^a^
17	Hydroxyluteolin/Quercetin-*O*-hexoside	I	97.7 ± 14.2 ^a^	126.7 ± 13.3 ^a^	139.8 ± 1.6 ^a^	131.8 ± 9.2 ^a^
18	Rosmarinic acid-*O*-hexoside	J	1050.2 ± 279.2 ^a^	834.7 ± 200.3 ^a^	1047.5 ± 99.2 ^a^	917.1 ± 96.2 ^a^
19	Luteolin-*O*-rutinoside	K	679.4 ± 20.9 ^a^	370.3 ± 83.3 ^ab^	348.5 ± 66.1 ^b^	532.5 ± 28.8 ^ab^
20	Luteolin/Scutellarein-*O*-glucuronide (I)	I	1164.0 ± 37.9 ^a^	627.2 ± 86.8 ^b^	591.3 ± 123.1 ^b^	893.9 ± 41.2 ^ab^
21	Luteolin-7-*O*-glucoside	I	248.4 ± 8.7 ^a^	243.9 ± 12.7 ^a^	205.0 ± 8.7 ^ab^	184.6 ± 3.4 ^b^
22	Nepitrin	I	2492.5 ± 14.8 ^a^	1804.6 ± 70.6 ^b^	1765.4 ± 124.4 ^b^	2061.6 ± 40.8 ^b^
23	Apigenin-*O*-rutinoside	K	136.4 ± 1.2 ^a^	65.0 ± 8.4 ^b^	53.4 ± 11.4 ^b^	63.3 ± 1.7 ^b^
24	Rosmarinic acid	J	47,541.6 ± 10,449.0 ^a^	42,038.5 ± 4795.7 ^a^	50,352.6 ± 3949.0 ^a^	46,825.6 ± 77.7 ^a^
25	Hesperidin	L	964.8 ± 58.4 ^a^	869.2 ± 3.3 ^a^	713.9 ± 84.5 ^a^	859.9 ± 36.7 ^a^
26	Homoplantaginin	I	1729.9 ± 22.5 ^a^	875.1 ± 69.8 ^b^	900.4 ± 108.0 ^b^	1011.2 ± 19.5 ^b^
27	Luteolin/Scutellarein-*O*-glucuronide (II)	I	1288.3 ± 277.3 ^a^	1204.7 ± 14.1 ^a^	725.7 ± 157.2 ^a^	1434.7 ± 22.5 ^a^
28	Tetrahydroxyflavone-*O*-acetylglucuronide (I)	I	301.3 ± 39.7 ^a^	224.6 ± 9.7 ^a^	178.8 ± 39.0 ^a^	311.6 ± 22.1 ^a^
29	Tetrahydroxyflavone-*O*-acetylglucuronide (II)	I	267.7 ± 48.0 ^a^	253.7 ± 0.6 ^a^	162.0 ± 35.5 ^a^	310.2 ± 12.1 ^a^
30	Tetrahydroxyflavone-*O*-acetylglucuronide (III)	I	223.9 ± 47.5 ^a^	244.7 ± 7.9 ^a^	139.2 ± 23.8 ^a^	253.8 ± 2.4 ^a^
31	(Epi)(iso)Rosmanol (I)	M	802.7 ± 258.7 ^b^	12,960.9 ± 1928.9 ^a^	15,272.0 ± 922.7 ^a^	15,975.9 ± 1377.5 ^a^
32	Rosmanol derivative	M	4941.4 ± 334.6 ^a^	3604.9 ± 1072.5 ^a^	6538.5 ± 1189.6 ^a^	6249.1 ± 578.8 ^a^
33	(Epi)(iso)Rosmanol (II)	M	28,364.3 ± 194.3 ^a^	21,466.6 ± 3748.7 ^a^	24,849.2 ± 98.3 ^a^	22,977.6 ± 1608.3 ^a^
34	Cirsimaritin	N	32.1 ± 2.3 ^a^	32.1 ± 6.1 ^a^	43.6 ± 5.5 ^a^	41.1 ± 5.0 ^a^
35	(Epi)(iso)Rosmanol (III)	M	13,647.7 ± 2013.1 ^a^	2698.7 ± 267.9 ^b^	5604.5 ± 456.7 ^b^	5888.5 ± 235.3 ^b^
36	Carnosic acid isomer	M	1185.6 ± 55.0 ^a^	832.9 ± 63.0 ^b^	602.7 ± 52.3 ^b^	810.8 ± 37.1 ^b^
37	Genkwanin	O	0.7 ± 0.2 ^a^	1.7 ± 0.8 ^a^	1.8 ± 0.3 ^a^	1.1 ± 0.2 ^a^
38	Isocalolongic acid	M	1225.6 ± 145.0 ^b^	9520.2 ± 1416.1 ^a^	11,964.3 ± 511.7 ^a^	7809.5 ± 1487.5 ^a^
39	Rosmadial/Safficinolide (I)	M	10,479.4 ± 2532.0 ^a^	733.9 ± 57.8 ^b^	1358.8 ± 208.3 ^b^	2384.1 ± 6.5 ^b^
40	Epirosmanol methyl ether	M	999.0 ± 30.4 ^a^	126.5 ± 8.6 ^b^	278.2 ± 53.3 ^b^	826.5 ± 0.1 ^a^
41	Carnosol	M	12,227.1 ± 2277.0 ^c^	23,022.8 ± 260.8 ^b^	34,392.3 ± 410.0 ^a^	27,136.5 ± 2089.0 ^ab^
42	Rosmadial/Safficinolide (II)	M	1506.4 ± 125.5 ^a^	588.3 ± 18.2 ^b^	1707.7 ± 200.5 ^a^	1095.7 ± 38.7 ^ab^
43	Rosmaridiphenol	M	707.7 ± 121.2 ^a^	235.7 ± 37.9 ^b^	546.2 ± 88.9 ^ab^	407.9 ± 5.5 ^ab^
44	Carnosic acid	M	90.1 ± 38.2 ^b^	456.7 ± 38.1 ^a^	580.4 ± 22.3 ^a^	183.0 ± 8.7 ^b^
Total			154,094.7	141,602.1	180,129.0	168,924.0
			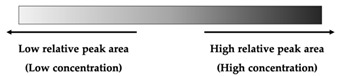			
** Total Phenolic acids**			51,813	46,984	56,002	52,398
** **Total Hydroxybenzoic acids			2390	2939	3121	3403
** **Total Hydroxycinnamic acids			49,423	44,046	52,881	48,995
** Total Flavonoids**			24,979	17,518	19,889	24,344
** **Total Flavan-3-ols			15,352	10,574	13,921	16,253
** **Total Flavones			8662	6074	5255	7231
** **Total Flavanones			965	869	714	860
** Total Phenolic diterpenes**			74,952	66,728	91,731	83,936
** Total Hydroxybenzaldehydes**			1126	852	453	437
** Total Coumarins**			n.d.	n.d.	90	n.d.
** Total Pyranochromanones**			1226	9520	11,964	7810

For each commercial brand, the concentrations are expressed as mean ± standard deviation (ng/mL of infusion) of the infusions prepared in triplicate. Within each line, different letters represent significant differences between commercial brands, at *p* < 0.05. n.d., not detected. *, Reference standard used for quantification or semi-quantification: A: Protocatechuic acid, B: Syringic acid, C: Vanillic acid, D: 4-hydroxybenzoic acid, E: *p*-Coumaric acid, F: Caffeic acid, G: Chlorogenic acid, H: Catechin, I: Luteolin-7-*O*-glucoside, J: Rosmarinic acid, K: Rutin, L: Hesperidin, M: Carnosic acid, N: Hesperetin, O: Genkwanin.

**Table 5:** Free amino acids (ng/mL) of rosemary infusions from different commercial brands, detected and quantified by RP-HPLC-FLD.

Free Amino Acid	Commercial Brand			
	A	B	C	D
Aspartic acid	n.d.	n.d.	n.d.	n.d.
Glutamic acid	n.d.	n.d.	n.d.	n.d.
Asparagine	tr	1.28 ± 0.00 ^b^	1.34 ± 0.00 ^a^	n.d.
Serine	tr	n.d.	n.d.	n.d.
Glutamine	n.d.	n.d.	n.d.	n.d.
Histidine	n.d.	n.d.	n.d.	n.d.
Glycine	n.d.	n.d.	n.d.	n.d.
Threonine	n.d.	n.d.	1.27 ± 0.02 ^a^	1.27 ± 0.01 ^a^
Arginine	n.d.	n.d.	n.d.	n.d.
Alanine	1.03 ± 0.01 ^a^	1.01 ± 0.03 ^ab^	1.00 ± 0.01 ^ab^	0.97 ± 0.00 ^b^
Tyrosine	n.d.	n.d.	0.02 ± 0.00	n.d.
Valine	n.d.	n.d.	n.d.	n.d.
Methionine	n.d.	n.d.	n.d.	n.d.
Tryptophan	n.d.	n.d.	n.d.	n.d.
Phenylalanine	0.57 ± 0.06 ^b^	0.68 ± 0.01 ^b^	0.89 ± 0.08 ^a^	0.95 ± 0.03 ^a^
Isoleucine	0.68 ± 0.02 ^b^	0.76 ± 0.02 ^b^	0.87 ± 0.04 ^a^	0.95 ± 0.02 ^a^
Leucine	n.d.	n.d.	n.d.	n.d.
Lysine	n.d.	n.d.	n.d.	n.d.
Hydroxyproline	n.d.	n.d.	n.d.	n.d.
Proline	0.20 ± 0.01 ^a^	0.18 ± 0.02 ^a^	0.19 ± 0.02 ^a^	0.17 ± 0.01 ^a^

The results are expressed as mean ± standard deviation (infusions prepared in triplicate). Within each line, different letters symbolize significant differences between commercial brands (*p* < 0.05). n.d., not detected; tr, traces.

**Table 6:** Pearson correlations obtained between the different spectrophotometric assays.

	DPPH^•^ Inhibition	FRAP	ORAC	Total Phenolic Content	Total Flavonoids Content
DPPH^•^ inhibition	1	−0.469	−0.116	−0.447	−0.573
FRAP	−0.469	1	0.647 *	0.934 **	0.917 **
ORAC	−0.116	0.647 *	1	0.552	0.562
Total phenolic content	−0.447	0.934 **	0.552	1	0.943 **
Total flavonoids content	−0.573	0.917 **	0.562	0.943 **	1

**, Correlation is significant at the 0.01 level (2-tailed); *, Correlation is significant at the 0.05 level (2-tailed). DPPH^•^, 1,1-diphenyl-2-picrylhydrazyl radical; FRAP, Ferric reducing antioxidant power; ORAC, Oxygen radical absorbance capacity.
